# Improved and Robust Detection of Cell Nuclei from Four Dimensional Fluorescence Images

**DOI:** 10.1371/journal.pone.0101891

**Published:** 2014-07-14

**Authors:** Md. Khayrul Bashar, Kazuo Yamagata, Tetsuya J. Kobayashi

**Affiliations:** 1 Leading Graduate School Promotion Center, Ochanomizu University, Tokyo, Japan; 2 Research Institute for Microbial Diseases, Osaka University, Osaka, Japan; 3 Institute of Industrial Science, The University of Tokyo, Tokyo, Japan; Pennington Biomedical Research Center/LSU, United States of America

## Abstract

Segmentation-free direct methods are quite efficient for automated nuclei extraction from high dimensional images. A few such methods do exist but most of them do not ensure algorithmic robustness to parameter and noise variations. In this research, we propose a method based on multiscale adaptive filtering for efficient and robust detection of nuclei centroids from four dimensional (4D) fluorescence images. A temporal feedback mechanism is employed between the enhancement and the initial detection steps of a typical direct method. We estimate the minimum and maximum nuclei diameters from the previous frame and feed back them as filter lengths for multiscale enhancement of the current frame. A radial intensity-gradient function is optimized at positions of initial centroids to estimate all nuclei diameters. This procedure continues for processing subsequent images in the sequence. Above mechanism thus ensures proper enhancement by automated estimation of major parameters. This brings robustness and safeguards the system against additive noises and effects from wrong parameters. Later, the method and its single-scale variant are simplified for further reduction of parameters. The proposed method is then extended for nuclei volume segmentation. The same optimization technique is applied to final centroid positions of the enhanced image and the estimated diameters are projected onto the binary candidate regions to segment nuclei volumes.Our method is finally integrated with a simple sequential tracking approach to establish nuclear trajectories in the 4D space. Experimental evaluations with five image-sequences (each having 271 3D sequential images) corresponding to five different mouse embryos show promising performances of our methods in terms of nuclear detection, segmentation, and tracking. A detail analysis with a sub-sequence of 101 3D images from an embryo reveals that the proposed method can improve the nuclei detection accuracy by 9 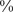 over the previous methods, which used inappropriate large valued parameters. Results also confirm that the proposed method and its variants achieve high detection accuracies (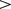 98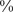 mean F-measure) irrespective of the large variations of filter parameters and noise levels.

## Introduction

The development of time-lapse imaging technique using fluorescent proteins (e.g., green fluorescence protein (GFP)) creates ample opportunities of recording optically sectioned images of biological samples. These images can be used to uncover complicated biological processes like embryogenesis, endocytosis or fusion (during viral infections), and disease (e.g., cancer) spreading [1]–[4]. Mouse embryogenesis involves many biological processes (e.g., cellular division, differentiation, and apoptosis) that can be unveiled through studying cellular dynamics. However, understanding cell dynamics requires the accurate extraction and tracking of cell nuclei over high dimensional space and time [5]. Our objective is therefore to develop computational technique for automated extraction of nuclear information based on image analysis. Given appropriate temporal resolution, individual cells can be followed over time, providing a continuous recording of proliferation, differentiation, and morphogenesis. However, exploring above information from 4D time-series is not trivial due to imaging limitations and the non-uniformity of the responses of fluorescence probes, especially when nuclei get closer at higher developmental stages [6].

To solve the problem for accurate nuclei detection, two main research streams can be found. First one performs nuclei segmentation followed by centroid extraction. Methods in this stream integrate simple threshold-based results with other image processing methods like morphological operations, mode finding, watershed, and level set to segment nuclei in the presence of noise, uneven contrast, and juxtaposed nuclei [7]–[16]. However, most such methods are either manual or very specialized to particular organisms and/or imaging techniques. Schnabel *et al.* [11] proposed a software implementing a 3D interactive method for manually identifying cell-nuclei of *C. elegans*, while Parfitt *et al.* [12] followed the same technique to establish lineage allocation in the early mouse embryo. Although these methods claim improved results, their manual cell marking procedure is time-consuming and includes subjective errors. Keller *et al.* [13] adopted specially designed digital scanned laser light sheet fluorescence microscopy (DSLM) to study embryogenesis of zebra fish. Good segmentation results were achieved due to high signal to noise ratio (SNR) and spatial resolution of the DSLM images. However, image signatures of a mouse embryo are quite different from those of a zebra-fish because of the differences in the tissue organization. In case of the mouse embryo, cells grow inside the non-transparent tissue volume, whereas they (i.e., cells) grow in a thin tissue-layer on the zebra-fish embryo surface that encloses transparent materials. Therefore, the methods that are specific to certain organisms (e.g., zebra fish or C.elegans) and/or special imaging techniques (e.g., DSLM) may not be applicable to the usual confocal images for mouse embryogenesis.

The second research stream [14]–[21] applies matched filtering for the enhancement of object-intensity distribution that facilitates computing nuclei centers as local maxima. Although the methods in this stream have the potentials of increased performance against juxtaposed cells, a little attention is observed so far. Byun *et al.* applied inverse Laplacian of Gaussian (LoG) filter to the enhanced feline retina images. The length of the filter was considered proportional to the empirically fixed average nuclear diameter [14]. Bao *et al.* [15] proposed a similar enhancement procedure using cube filter, whose length was also considered as the mean diameter of the available nuclei at each time-point. However, the fixed-length filter does not provide accurate spatial enhancement. A. Santella *et al.* applied difference of Gaussian (DoG) filtering for enhancing 3D images, but this method does not perform adaptive smoothing, usually required for better enhancement and detection [16]. J. Han *et al.* proposed a multiscale iterative radial voting technique for nuclear seed estimation [17]. Although this method is claimed to have robustness against noise, it was tested only with 3D images having relatively high voxel resolution (0.15 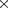 0.15 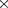 0.75 µm). Moreover, the method did not discuss about parameter optimization for varying nuclei sizes. Bashar *et al.* proposed a multiscale spatial enhancement method for nuclei detection [18]. Although good results were achieved with manually selected parameters (i.e., the minimum and maximum filtering lengths), an inaccurate parameter selection may lead to poor detection accuracy. Since nuclei sizes vary over space and time, temporal adaptation is also important. Recently, Bashar *el al.* [19]–[21] proposed spatio-temporal techniques, which estimate the minimum and the maximum filter lengths based on the normalized-volume-ratio (NVR) of nuclear objects between the current and all of the previous frames. These approaches improve the detection accuracy 2 to 3 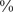 over the previous method [18]. However, computed filter lengths are less accurate, because NVR includes background pixels during the computation of mean object volume. Since more automation with lesser manual parameters and the robustness to the variations of the enhancement and noise parameters are important biological demands, we propose a multiscale adaptive method for the robust detection of cell nuclei from 4D fluorescence images. The contributions of our paper are as follows:

- An efficient segmentation-free direct nuclei detection method is proposed. This method employs multiscale spatio-temporal adaptive enhancement using Gaussian filtering, which can create more homogeneous object-structures for efficient and robust nuclei detection with reduced parameters. A feedback mechanism, based on optimizing a radial gradient function, is designed to achieve above robustness with improved detection accuracy. A simplified form of the proposed method is suggested for more parameter reduction. Single-scale versions of the above two methods are also analyzed with respect to nuclear detection.
- A centroid-driven segmentation method is developed for nuclear volume extraction. This method integrates the extracted centroid information and the mentioned feedback mechanism to segment spherical nuclei volumes even when the cells are juxtaposed. This method can therefore be used for separating clustered nuclei especially at high developing stages.
- Detail experimental evaluation and comparison are performed with special focus on the robustness against parameter and noise variations.
- A simple tracking method using distance optimization is also discussed. This method associates estimated centroids between two consecutive 3D frames based on nearest neighbor principle. Preliminary evaluation in the fixed population case indicates promising performance of the method.

The rest of the paper is organized as follows. In the “Proposed Method” section, a detail description of the proposed method is given including its extended feature on the spatio-temporal adaptiveness to the robust nuclei detection. The system used for imaging mouse embryos at early developing stages, the characteristics of imaging data, and the procedure for ground-truth formation are explained in the “Imaging System and Data” section. The section entitled “Experimental Evaluation” describes the evaluation and comparative performance of our method using imaging data including the discussion on the obtained results. Finally, our work is summarized in the “Conclusion” section.

## Proposed Method

**Figure 1:**
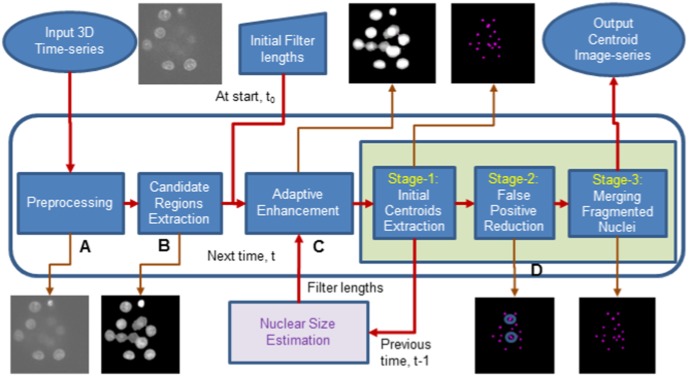
Block diagram of the proposed adaptive method.

The proposed method extracts nuclei centroids from every three dimensional image corresponding to each discrete time point. An overview of the proposed method is shown in Fig. 1.

### Preprocessing

Fluorescence images suffer from a set of disturbances induced by confocal microscopy systems: blur effects induced by the point spread function of optical setup, Poisson noise that arises from photon counting process, and additive Gaussian shot noise. Moreover, nuclear signal is heterogeneous due to heterogeneous structures of chromatin, yielding bright spots within the nuclei regions. Incorrect parameter settings may induce partial occlusion of image objects, including unexpected discrete noises. To reduce noise effects, we first apply a 3D median filter of 5
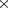
5
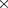
3 pixels to remove bright spots or other impulsive noise in the data. A cubic interpolation is then performed in the z-direction to obtain approximately isotropic voxels. Above two operations generate a preprocessed image, denoted as 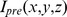.

### Candidate Regions Extraction

Candidate regions of interest approximately encompass nuclei regions in the image. These regions were segmented by roughly removing background regions. The use of candidate regions brings two important benefits. Firstly, it reduces the risk of detecting strong noise peaks (if any) in the later steps. Secondly, it saves processing time for large volume biological images. In our research, we apply Otsu global threshold method [22], which exploits the bi-modality of the image histogram to obtain candidate binary masks from the preprocessed image. Finally, the candidate region image, 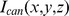, is obtained by retaining the contents of the pre-processed image corresponding to the binary regions.

### Spatio-temporal Adaptive Enhancement

Image enhancement is an important step for the segmentation-free direct extraction of cell nuclei. In general, this process smooths micro-structures inside object regions and assists in finding nuclei centroids using simpler techniques. Cell nuclei typically vary in sizes and shapes over space and time, especially during mouse embryo development in the early stages. A spatio-temporal adaptive enhancement is therefore effective for our research. Our previous method employs multiscale enhancement using fixed filtering parameters over time [18]. An interactive selection of fixed parameters can produce fairly good results. However, the wrong selection of parameters may cause detrimental effects on the final outcome. Data-driven adaptive enhancement is therefore necessary to minimize the remaining noise components in the candidate-region image. This step ensures proper smoothing the object surfaces in the 3D candidate-region image and makes them suitable for subsequent processing. Three important benefits that can be achieved from adaptive filtering are: (i) spatio-temporal adaptive enhancement ensures high detection accuracy; (ii) it brings robustness to the detection method against variations of initial filtering parameters; (iii) it also suppress noise and provides more automation to the system through parameter reduction. If 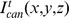 be the 3D candidate region image, and 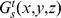 be the base enhancement filter at scale index 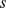, the enhanced image at time 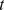 can be computed using discrete convolution:
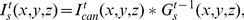
(1)where 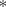 represents the convolution operator. The time index in 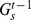 indicates that the filter kernel is computed from the previous frame to obtain the enhanced image of the current frame, i.e., 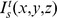. We choose 3D Gaussian function as the base filter that can be given by
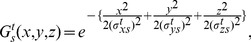
(2)where 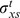, 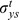, and 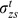 are the spread parameters of the Gaussian. In our research, separable one dimensional discrete Gaussian filters (e.g., 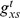, 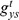, and 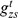) were used for implementing discrete convolution in Eq. 1. If the finite length of the filter along the x direction is given by 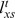, we can relate it to the spread parameter by 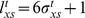. With this length, 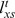, the 1D Gaussian filter is defined by
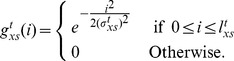



Similar equations can be defined for 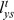, 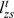, 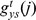, and 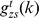, respectively. These filter lengths (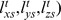) are the required filter parameters to be estimated from the image. In our research, we use isotropic filters for which one dimensional finite filter lengths are given by 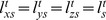. Therefore, the spread parameter for the isotropic filter can be obtained by 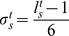.

#### Multiscale Adaptive Filtering

Multiscale filtering is an appropriate choice, when nuclei have size variations with respect to time and space. Bashar *et al.* proposed such an approach, where the minimum and maximum filter lengths (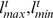) required for spatial adaptation is computed from the current and all of the previous time-point images [19]–[21]. Normalized volume ratio of the candidate object pixels was used to obtain above lengths. However, the inclusion of background pixels in the candidate regions especially in the case of touching nuclei limits the improvement of the detection results by 2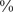 on average. In this study, we estimate the above lengths based on the extracted nuclei objects through optimization as detailed in the section *Nuclear Size Optimization*. In a single-embryo image, if 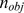 be the total nuclei and 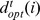 is the optimal diameter (See Eq. 15) for the 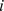
th nucleus, we can obtain the minimum and maximum object sizes by
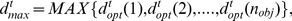
(3)

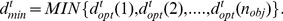
(4)


Once the maximum and minimum object sizes were known, we can compute 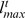 and 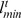 by
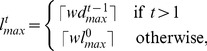
(5)

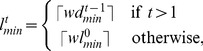
(6)where 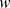 is the weighting factor, varying from 0 to 1. It is chosen 1.0 for the selected Gaussian filter. Note that 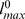, 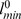 are the maximum and the minimum filter lengths, used for processing the first frame in the sequence. At any time-point t, if 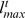 and 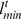 are known, we can obtain multiple filter lengths for the spatial optimization using
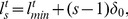
(7)where 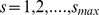; 
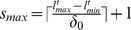
, and 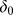 is the fixed incremental length. With the above lengths, multiple filters are applied at every pixel of the 3D image using Eq. 1 and the resulting responses are maximized by
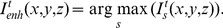
(8)


Spatio-temporal adaptation described above provides stable nuclei detection, which is insensitive to the initial filter parameters.

#### Single-scale Adaptive Filtering

If nuclei in a single time-point 3D image have very small variations in sizes, single-scale temporal adaptation is an alternate option. In the single case, the enhanced image in Eq. 1 can be given by
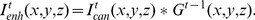
(9)


Once again, we select Gaussian function as our base filter, whose spread parameter (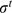) in the separable and isotropic case, can be related to the finite length parameter (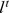) by 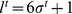. This length should corresponds to the mean size of the nuclei for the adaptive enhancement. In our study, an initial value (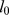) of this parameter has been chosen empirically. Once we have 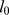 at hand, we can run the proposed size optimization step (Please refer to the section *Temporal Feedback Mechanism*) to estimate the average object diameter (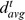) from all nuclei of the image at time 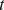. If a single-embryo image at time 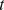 have 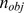 nuclei and 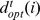 is the optimal diameter (See Eq. 15) for the i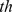 nucleus, we can compute
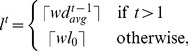
(10)where 
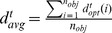
 and 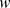 is the weight factor as described before.

### Centroid Extraction

Three stages of processing are performed for centroid extraction. **Stage-1** performs the computation of local maxima and generates maxima clusters using threshold on the characteristic ratio, which represents the percentage of lower intensity voxels from the central voxel in a local neighborhood. Connected component labeling followed by averaging is performed to obtain initial centroids. **Stage-2** refines Stage-1 results by analyzing the convexity of the intensity profiles in three orthogonal directions. Previous method used a fixed length during the selection of orthogonal intensity profiles [18]. Profile lengths in the previous method were assumed to be less than 50 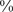 of the diameter of the largest nucleus in the image sequence. However, the proposed method automatically estimates the mean object diameter from the 3D image and uses it as the profile length for the next image in the sequence. **Stage-3** merges fragmented nuclei (if any) in the Stage-2 results. Note that above three stages involve three threshold parameters, i.e., the ratio, shape, and distance parameters (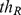, 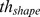, 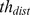), respectively. Please refer to our previous work [18] for the detail of these stages. However, we assign 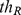 to 100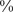 in this research, because our proposed method reduces inhomogeneity in the object intensity distribution and facilitates selecting threshold value (Please refer to the next section for detail analysis).

### Temporal Feedback Mechanism

This step estimates filter parameters from the current image and sends them to process the next image in the sequence. Such procedure performs the proper enhancement based on the sizes of available objects in the image. In this study, nuclei are assumed to have spherical shape and their diameters are considered as parameters for multiscale enhancement of images. Nuclear diameters are estimated using the enhanced image. We introduce a new objective function 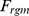, called **radial gradient magnitude (RGM)**, to estimate nuclei diameters at each position of Stage-1 centroids. This function is defined as the absolute difference between two one dimensional functions, computed from the 3D enhanced candidate region image. The first signal represents the spatial variations of mean intensities in the concentric spherical volumes that are gradually increasing in radius from each center. The second is the mean intensities of spherical annular regions between two consecutive radius-pairs starting from the smallest sphere of 1 pixel radius. If 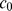 is the centroid position of a Stage-1 nucleus, RGM and the related functions can be defined by
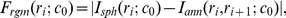
(11)where
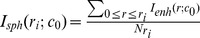
(12)

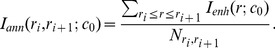
(13)


**Figure 2:**
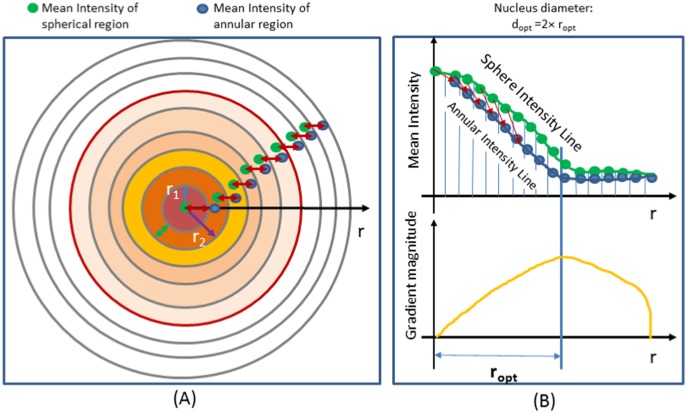
Computation of Radial Gradient Magnitude (RGM). Schematic diagrams for (A) computing 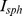 and 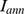, and (B) showing the spatial variations of RGM values.

In the above equations, 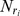 and 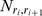 are the total voxels in the spherical and annular regions bounded by 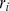 and 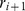 as shown in Fig. 2(A) and
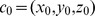


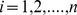


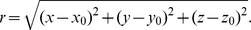



**Figure 3:**
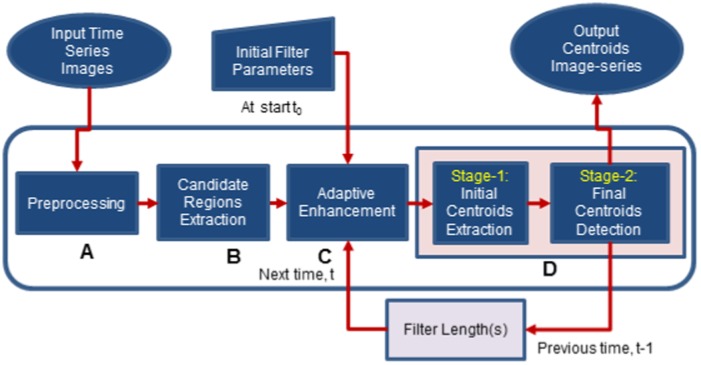
Block diagram of Variant-2 and Variant-3 of proposed method.

Since 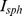 corresponds to the mean intensities within the concentric spheres, its values fall slowly than those in the annular regions, 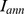 as shown in Fig. 2(B). As a result, RGM increases gradually from the center to the object boundary, where it becomes maximum and then starts falling due to approximately constant values of 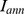 until reaching to the zero value (See Fig. 3(B)). The inner radius at the maximum RGM value can be regarded as the optimal radius of the nucleus. This is achieved by maximizing the objective function as in Eq. 14.
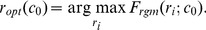
(14)


Note that the radius 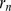 denotes the approximate object boundary surface, while the radius 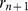 indicates the surface in the background region. For notational convenience, the optimal radius for the 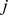
th nucleus at time 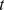 is denoted by 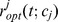. Finally, the object size, i.e., the optimal diameter of the extracted nucleus is obtained by
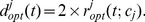
(15)


Stage-1 (Please refer to the section Centroid Extraction may contain false centroids due to remaining noises after adaptive enhancement. However, the estimated diameters for such objects may be very small. We therefore eliminate spurious objects before computing final parameters. We empirically choose a minimum size threshold (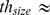 10 pixels), which is less than the half of the largest nucleus diameter in the recorded images. The maximum, minimum, and the average diameters of the Stage-1 nuclei are used as feedback parameters to adaptively enhance the next sequential image.

### Other Variants of Proposed Method

Variant-1 is the single-scale version of the proposed method. In this variation, the enhancement of the candidate region image is done using single scale adaptive filtering. Variant-2 and Variant-3 are the simplified versions of the proposed method and its single-scale variant. These methods are aimed for the parameter reduction. Block diagram of the simplified method is shown in Fig. 3. The preprocessed image undergoes multiscale (or single-scale) adaptive enhancement. Centroid extraction here consists of two stages. Stage-1 extracts the initial nuclei centroids as before, while the last stage (Stage-2) employs the feedback mechanism (as explained above)for detecting final centroids. This stage estimates nuclear diameters and centroids by size optimization. A size threshold (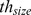) is applied as before to eliminate spurious objects. Note that the simplified method does not use Stage-2 and Stage-3 of the proposed method. As a result, the relevant parameters, i.e., 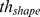 and 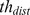 are also reduced.

### Centroid-driven Nuclear Volume Segmentation

**Figure 4:**
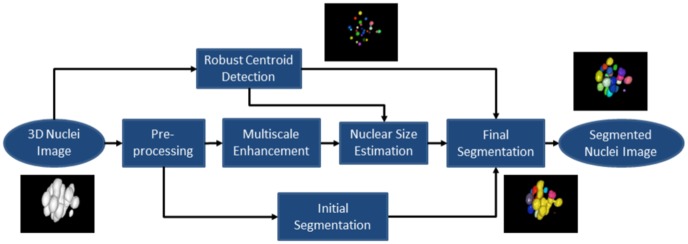
Block diagram of the method for Nuclear Volume Segmentation.

Proposed nuclei detection method can be integrated with a simple threshold technique for nuclear volume segmentation. It has an advantage of recovering approximate nuclear volumes from clustered nuclei at high developmental stage. Figure 4 shows the flow diagram for nuclear volume segmentation. The same optimization method (as in the section *Temporal Feedback Mechanism*) is used to estimate nuclear diameter at each final centroid position in the enhanced 3D image. This diameter along with the respective centroid is then projected onto the result (binary image) of a typical segmentation method. As results, the final segmentation of the nuclear volumes is achieved. This volume information can be associated with many applications including mitosis detection, object classification, and the object tracking.

### Computational Efficacy, Hardware, and Software

The proposed method is implemented using Microsoft Visual Studio 2008 on the windows platform. Most of the source codes are written using visual C++ except few cases, where we use some functions from freely available image processing library [26]. Volume rendered images are prepared using non-free software “PLUTO” [25]. Codes for plotting tracking diagrams are written using commercially available MATLAB software. Source code is not open at the moment. All tests are conducted using a windows PC having an Intel(R) Core(TM) i7 CPU 3.20GHz with 8GB RAM. Proposed robust detection program takes about 0.05 minutes to process a single frame, i.e., a 3D single embryo image of size 103 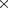 103 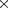 102.

## Imaging System and Data

### Imaging System

Mouse embryo images were captured by Cell Voyager (CV1000) microscope system integrated with Nipkow disc confocal system, (Yokogawa Electric Corporation, Japan) with a 20× UPlan Apo NA = 0.8. Two main features of our microscopy system are (i) the ability of eliminating out-of-focus blur by confocal image capture and (ii) the low photo-toxicity due to lower incident light power per unit volume of the specimen. The reduction of the light power is achieved by segregating laser beam using multiple pinholes of the Nipkow disk. An mRNA injection based technique is applied to image mouse embryo cultured by *in-vitro* fertilization (IVF) [23], [24]. This technique consists of mRNA synthesis via *in vitro* transcription of plasmid encoding a fluorescent fusion protein following microinjection into the oocyte cytoplasm and subsequent observation using fluorescence microscopy. The fertilized oocytes were injected at the anaphase/telophase stage with 5 ng/µL of mRNA encoding histone H2B-mRFP1, so that cell nuclei and chromosomes were stained. An excitation light of 561 nm wavelength was applied at 15 minutes interval for about 70 hours, which approximately covers from 1-cell to the early blastocyst stage of mouse development.

#### Ethics Statement

Note that all of the animal experiments were performed with the approval of the Animal Care and Use Committee of Osaka University, Osaka, Japan.

### Imaging Data

**Figure 5:**
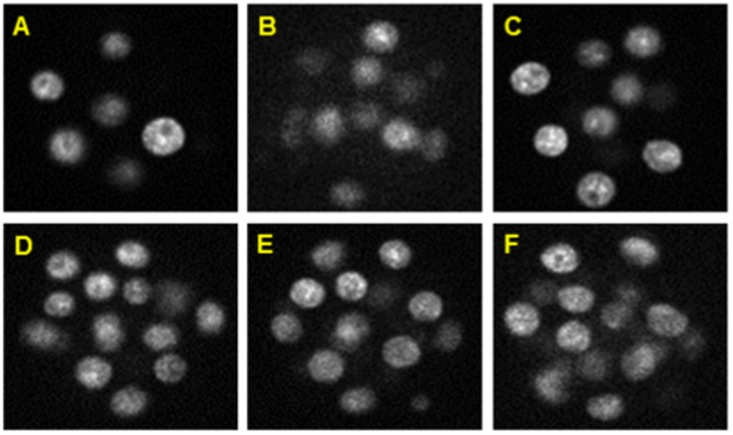
Contrast-enhanced 2D slices from our image dataset. Sample images with time-point and z-slice pairs at (A) (t25, 18), (B) (t34, 35), (C) (t45, 22), (D) (t86, 22), (E) (t91, 24), and (F) (t98, 29). Each image has dimension: 103 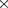 103 pixels and has voxel resolution: 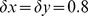 and 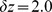 µm.

The original video consists of 271 time-points with 13821 fluorescence 2D images, each of size 512 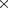 512 pixels. Each of the original pixels has resolution of 0.8 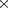 0.8 µm^2^ in the x-, and y-directions. Temporal resolution was 15 minutes, while the number of z-slices was 51 with 2 µm interval. Original dataset contains an image-sequence of twelve early developing mouse embryos. In our research, we create five image-sequences for five single embryos containing approximately 2 to 32 cells. Each single time-point image in these sequences has a resolution of 103 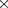 103 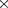 51 pixels. Each of the five derived sequences also contains 271 time-points, which can be denoted by s1 to s271. Two sub-sequences of 101 and 91 3D images (s170 to s271; s170 to s260) from the fifth embryo were used for detail study. For convenience, these two sets, each enclosing roughly 8 to 32 nuclei, were denoted by t1-t101 and t1-t91. Figure 5 shows a set of 2D contrast enhanced images from our datasets.

### Ground Truth Data

Nuclei centroid positions that can be measured from 3D images were considered as ground truth (GT) data. First of all, we generate preprocessed images by Gaussian smoothing and median filtering with appropriate interpolation. These images are then investigated using a visualization software PLUTO [25]. From each 3D image, we first generate a binary nuclei image using manual threshold in the software. 2D slices in the 3D binary images are then examined along the z-direction using mouse operation in PLUTO. For each nucleus, the slice containing the largest object cross-section gives the z-position, while the object-center in that slice gives the x- and y-coordinates for the nucleus. PLUTO GUI displays the image coordinates of each nucleus, when mouse is clicked at its center. The location of the nucleus center is fixed visually. The sliding button in the PLUTO screen indicates the z-slice number.

## Experimental Evaluation

In this section, we perform experiments on five 3D image sequences, obtained from five different mouse embryos. One hundred and one (101) 3D sequential images from an embryo have been used for detail quantitative analysis. Three well-known metrics are used for quantifying the results of nuclei detection [28].
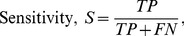
(16)

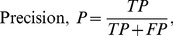
(17)

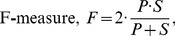
(18)where 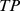 (i.e.,true positive), 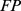 (i.e., false positive), and 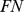 (false negative) represent the number of correctly detected, falsely detected, and the undetected nuclei, respectively.

### Computation of Evaluation Metrics

We compute above metrics at each time-point using the output binary-centroid image and the corresponding GT centroid positions for that image [18]. A local 3D spherical neighborhood (i.e., window) of 10 pixel radius (regarded as evaluation threshold, 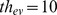) is centered at each GT position in the centroid image. In the expected case, this window will include at least one centroid as a correct detection. If it encloses more than one centroids, the one with the lowest distance is considered as the correct detection. In the worst case, it may include nothing. We can thus obtain a score of true positive (*TP*) from each 3D test image. Once 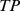 is counted, false positive (*FP*) and false negative (*FN*) are computed using 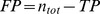 and 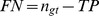, where 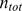 and 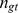 are the number of estimated and ground-truth centroids for the same test image.

### Results and Performance Analysis

Proposed method is evaluated by using datasets, described in the *Imaging Data* section. Following are the parameters [18] that we used for experimental evaluation of the proposed method and its variants: 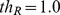, 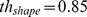, 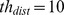 pixels, 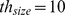, and 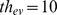 pixels. Note that the simplified variants of the proposed method (i.e., Variant-2 and Variant-3) do not require 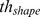 and 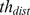 parameters. There are some parameters (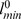 and 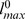 for multiscale, 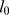 for single scale) that require to be selected only for the first frame during sequential processing. These were selected empirically along with miltiscale parameter 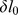, which remains fixed for processing whole image-sequence.

#### Qualitative Analysis

**Figure 6:**
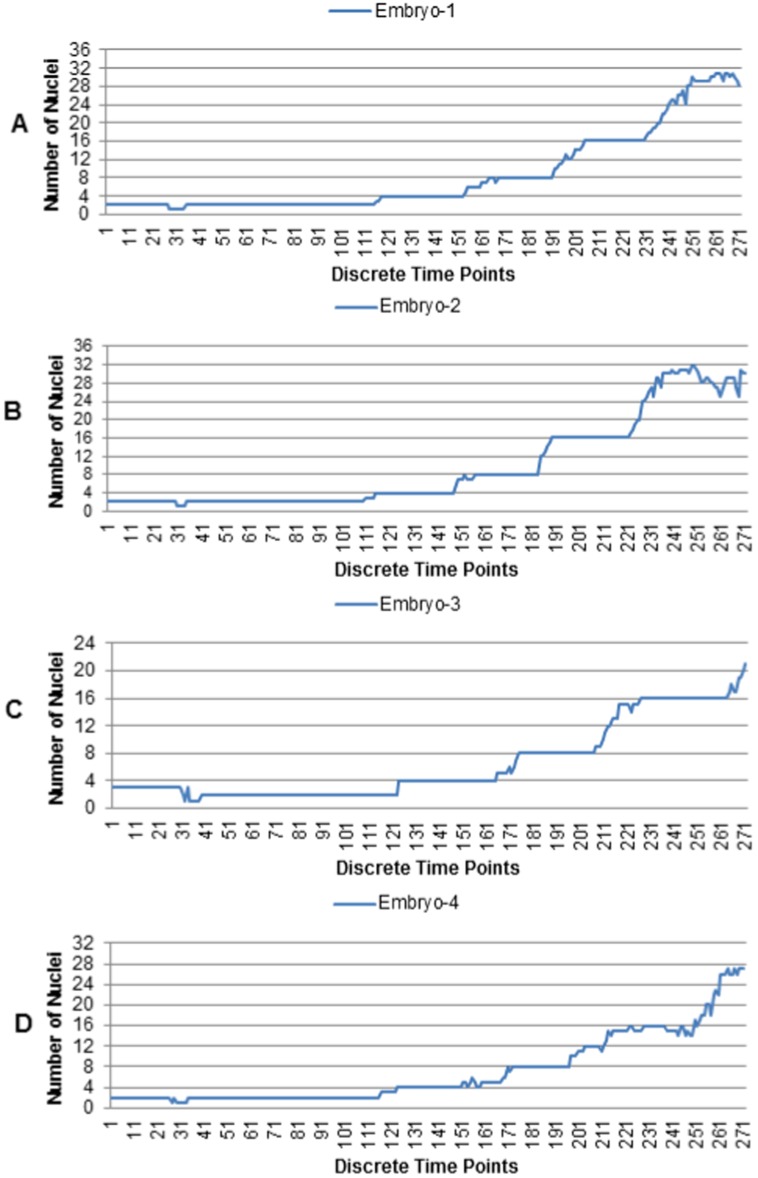
Estimated nuclei by the proposed method. Number of estimated nuclei for (A) embryo-1, (B) embryo-2, (C) embryo-3, and (D) embryo-4, respectively. Each embryo sequence consists of 271 images, i.e., s1 to s271, which contain nuclei from 2 to approximately 32 cell-stages.

Figure 6 shows the results of the estimated nuclei for four developing mouse embryos. Image sequences in these embryos cover mouse development until early blastocyst stage. Each embryo shows a consistent development from 1-cell stage to approximately 32 cell stage. Note that the estimated nuclei at or before the first discontinuity (at around frame s32) in each graph represents pronuclei of the two sex cells (i.e., male spermatozoa and female oocyte) at the zygotic 1-cell stage. Therefore, one cell stage of fertilized cell is not distinguishable from confocal images. The beginning of two-cell stage starts at around s41 and then undergo systematic cell divisions through multiple developmental stages (e.g., 2–4, 4–8, 8–16, or 16–32 cells). Above figure shows almost constant populations at different developmental stages, providing an indication of healthy embryos. It also indicates the efficiency of the proposed detection method. Note that the transition periods gradually increase towards larger populations, implying the asymmetric cell divisions of the early developing embryos. Another observation is the more frequent ups and downs in the nuclear count graphs at higher developing stages (e.g., 32-cells). This behavior indicates the increasing difficulties in detecting clustered cells at larger populations in an automated way.

**Figure 7:**
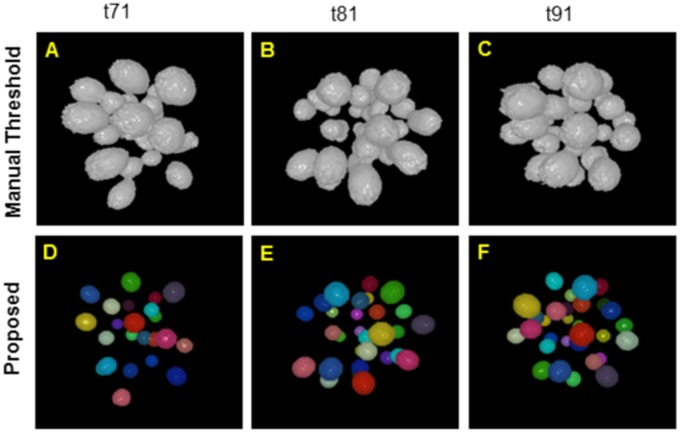
Estimated nuclei centroids at 16–32 cell-stage. Volume rendered views of nuclei for 3D images at (A) t71, (B) t81, and (C) t91, obtained by manual threshold and the corresponding (D-F) labeled centroid images, obtained by the proposed adaptive method. Initial filter lengths (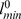, 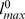) were set to their optimal values, i.e., (11, 31) pixels for multiscale Gaussian filtering.

**Figure 8:**
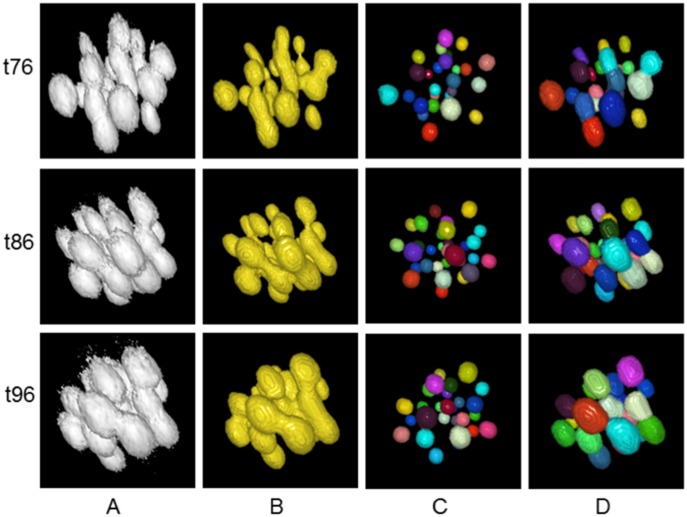
Clustered nuclei separation. Volume rendered views of the extracted nuclei centroids and volumes for 3D images, obtained by (A) manual threshold, (B) automated threshold, (C) the proposed centroid detection method, and (D) the proposed centroid-driven segmentation method. Sample images contain approximately 32 nuclei, which correspond to the developmental stages at high time-points i.e., at t76, t86, and t96 out of the sub-sequence of 101 images, used in our experiment.

Figure 7 shows volume rendered snapshots of the extracted nuclear centroids. The top row of Fig. 7 shows nuclei volumes, obtained by manual threshold, while the spherical color objects in the bottom row indicate the centroid detection ability of the proposed method, even when the nuclei are highly clustered. Figure 8 shows volume rendered views of the segmented nuclear volumes by manual (column A), typical (column B, [22]) and the proposed (column D) methods, respectively. Note that the proposed centroid-driven segmentation method successfully separates the clustered nuclei, even though there are lots of overlaps among closely located nuclei at high time-points (e.g., t76, t86, and t96).

**Figure 9:**
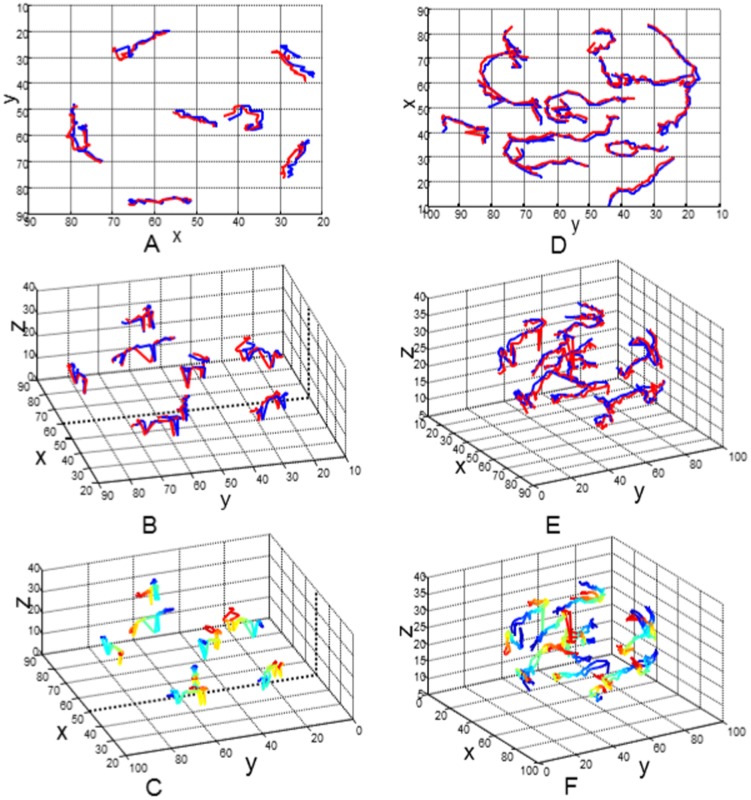
Nuclei tracking results at 8- and 16-cell stages of an embryo. Nuclear motion tracks in the (A, B, C) 8-cell and (D, E, F) 16-cell stages; Blue and red lines in the 2D projected space (A, D) and 3D space (B, E) indicate the estimated and ground truth nuclear tracks without explicit moving directions; Color gradients in the 3D space (C, F) indicate the directions through which cells move inside the embryo. Blue and red colors indicate the start and end points of the moving cells. Note that twenty two frames (i.e., t1 to t22) at the 8-cell stage and twenty six frames (i.e., t36 to t61) at 16-cell stage are used for track generation. The selected distance threshold (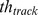) for the correspondence establishment was 25 pixels.

Results of nuclei detection have been applied for **tracking nuclei** using a simple frame by frame association method based on nearest neighbor searching technique [29]. Figure 9 shows the preliminary results at 8-cell (Fig.9 A–C) and 16-cell (Fig. 9 D–F) stages, respectively. Twenty two (i.e., t1–t22) and twenty six (i.e., t36–t61) frames in the 8-cell and 16-cell stages were used in this study. Cell movement patterns were shown in the 2D projection space (Fig. 9 (A, D)) as well as in the 3D space (Fig. 9 (B–C, E–F)). A good agreement between the estimated (blue lines) and the manually generated tracks (red lines) indicates the effectiveness of our tracking method. The color gradients of the tracklets in Fig. 9 (C, F) express the moving directions of nuclei within the embryo, where blue and red lines indicate the start and end points of their journey. However, small translational error between the estimated and the GT tracks do exists, which implies the subjective error of the ground truth data.

#### Quantitative Analysis

**Figure 10:**
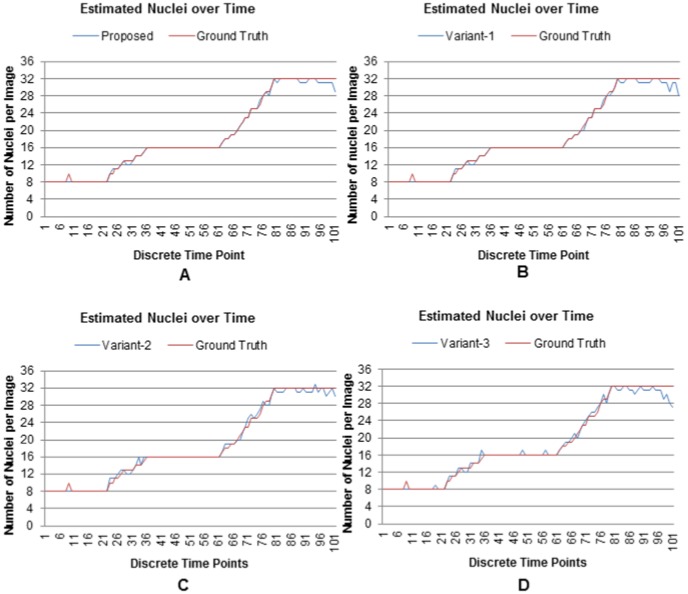
Nuclei detection results for the proposed method and its variants. Number of extracted nuclei by (A) the proposed, (B) Variant-1, (c) Variant-2, and (D) Variant-3 over time. Results include 101 sequential images from a developing embryo containing 8 to 32 cells.

Quantitative analysis is conducted using nuclei counts and F-measure metrics. We compute these metrics from 101 3D sequential images of a developing embryo that contains 8 to 32 cells. Figure 10 shows the results of the estimated nuclei over time. Blue lines in this figure show the estimated nuclei by the proposed method and its variations, while red lines show the ground truth nuclei. Correlations between blue and red curves roughly indicate the degree of correct detection by the proposed method and its variations. The proposed adaptive method showed clear superiority as the estimated lines closely follow the GT lines without much perturbations. The single scale version of the proposed method, i.e., Variant-1 also follows the GT curve except at the large population stage (e.g., 32 cells), where frequent ups and downs were observed. Variant-2 and Variant-3, which are the simplified versions of the proposed method, show more frequent oscillations in the estimated nuclei numbers that we observe throughout the nuclei-count curves. Note that there is spike in the ground truth at frame number 9 as shown in Fig. 10. This spike indicates ten nuclei at frame-9, while neighboring frames have constantly eight nuclei. In fact, the correct number will be eight, since the timing does not confirm any real cell division event. A close inspection however showed that it was a mistake happened during visual inspection.

**Figure 11:**
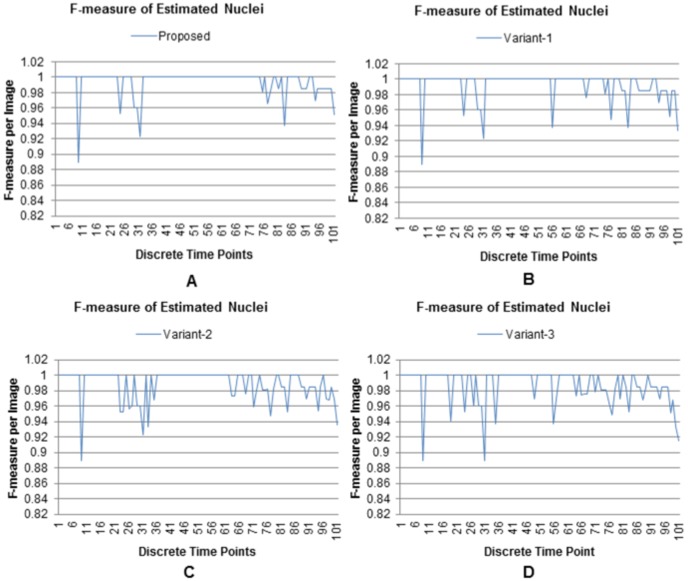
Performance comparison using F-measure. F-measure values, obtained by (A) the proposed, (B) Variant-1, (C) Variant-2, and (D) Variant-3 over time. Results include 101 3D sequential images from a developing embryo containing 8 to 32 cells.

Similar findings are reflected in the F-measure graphs (Figure 11), computed on the estimated nuclei. Very high values and less fluctuations of this metric, especially at high time-points, indicate the efficacy and stability of the proposed method and its single-scale variant. F-measure time-series for other variants (Variant-2 and Variant-3) show gradually increasing perturbations from multiscale to single-scale versions. These results are in congruence with the nuclear counting graphs in Fig. 10. One reason behind these observations is that the simplified methods (Variant-2 and Variant-3) use object-size threshold (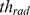), which sometimes removes small low-contrast objects with diameters less than 10 pixels (i.e., 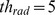 pixels). On the other hand, threshold operation in the proposed method (and its variant) might have a very small effects on the estimated filter lengths. However, such small perturbations do not affect the enhancement and subsequent detection steps in these methods. Both experiments also indicate that the multiscale enhancement based methods (proposed and Variant-2 Figs. 10 and 11 (A) and (C)) produce better results (in terms of nuclear counting and F-measure) than their single scale counterparts (Variant-1 and Variant-3 Figs. 10 and 11 (B) and (D)).

**Figure 12:**
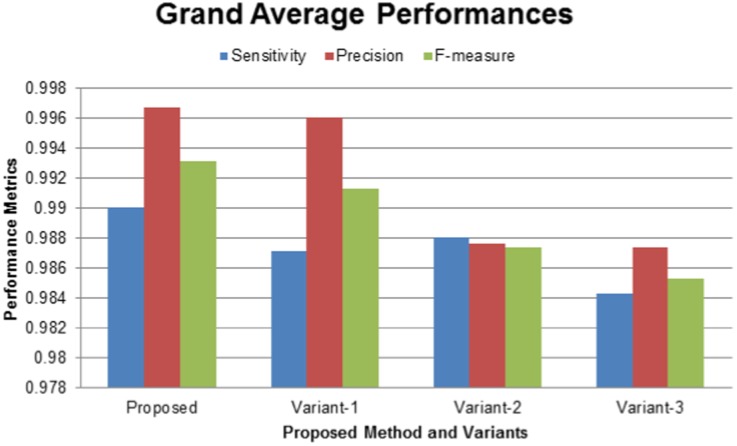
Performance comparison of the proposed method and its variants. Grand average scores of (Blue) sensitivity, (Red) precision, and (Green) F-measure, obtained by the proposed methods and its variants. Note that three metrics are averaged over temporal and parametric axes, where the temporal axis includes 101 sequential images, while the parametric axis includes ten different sets of filtering parameters.

The efficiency of the proposed method and its variants can be computed from the grand average values of the performance metrics as shown in Fig. 12. Note that the three estimated metrics (sensitivity, precision, and F-measure) are averaged over temporal (101 time-points) and parametric (Please refer to the next section) axes. On average, F-measure scores (green bars) of the methods in descending order are (i) Proposed (99.31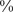), (ii) Variant-1 (99.13 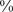), (iii) Variant-2 (98.74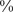), and (iv) Variant-3 (98.53 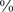). These results showed very competitive performances of the proposed method and its variants. Therefore, the use of the simplified methods may be an alternative, when users require handling a reduced set of threshold parameters. Note that multiscale enhancement-based methods (Proposed and Variant-2) showed slightly improved performances over their single scale counterparts (Variant-1 and Variant-3). This improvement was expected because nuclei sizes usually have intra- and inter-frame variations for which multiscale filtering usually works well.

**Figure 13:**
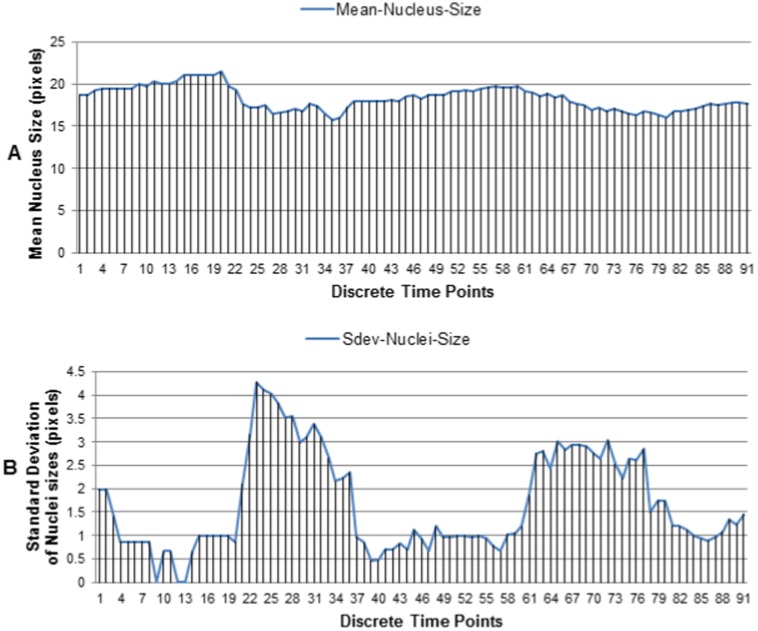
Estimated nuclei sizes by the proposed method. Temporal variations of (A) the mean nuclei diameters, and (B) the standard deviations of nuclei diameters. Results include 91 sequential 3D images from an embryo, containing 8 to 32 cells.

Estimating **nuclear volumes** is very important for various applications including tracking [15] and cellular phenotype analysis [4]. An experiment with 91 sequential 3D images (t1 to t91) is performed to estimate nuclear volumes applying the proposed method in Fig. 4. Estimated nuclear diameters and their variations are shown in Fig. 13. Two important observations are: (i) our datasets have small but smooth variations of average nuclei diameters over time (Fig. 13(A)); (ii) nuclei diameters have large variations during the occurrences of cell division events (i.e., t21–t35, t62–t79) compared to other times (Fig. 13 (B)). Therefore, standard deviation of the estimated nuclei sizes can be used to quantify gross transition periods of the cellular development in the multicellular embryos.

### Robustness Analysis and Performance Comparison

Robustness analysis is performed in two perspectives: (i) robustness to parameter variations and (ii) robustness to noise. In the first case, robustness is analyzed against the variations of filtering parameters for multiscale enhancement. In the second case, the robustness is justified against different noise levels. Comparative analysis is performed in terms of robustness consideration using sensitivity, precision, and F-measure metrics. Proposed method and its variants are compared with two methods: (i) multiscale-based non-adaptive nuclei detection method [18] and (ii) its single scale version. These methods are denoted as Previous (MSF) or Previous-1 and Previous (SSF) or Previous-2, where MSF and SSF stand for multiscale and single-scale filtering. Since Previous-1 method showed better performance compared to the method in [15], we will not consider it here for further comparison. Multiscale iterative radial voting is another recent method [17]. Due to lack of having publicly available source code, the detail one-to-one comparison with our proposed method is omitted here. However, this method showed 4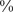 detection error with 144 mammosphere colony images, while our method shows (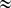 1 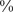) error with 101 mouse embryo images. Besides, this method was tested with high resolution images (0.15 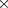 0.15 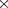 0.75 µm^3^), while our method is applied to relatively low resolution images (0.8 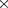 0.8 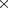 2.0 µm^3^).

#### Robustness to Parameter Variations

Expected outcome of the proposed method should be insensitive to the selection of the major parameters. Robustness to parameter variations is therefore an important consideration. Once again, sensitivity, precision, and F-measure metrics are used to evaluate parameter sensitivity. In our enhancement-based method, we consider single/multiscale filtering parameters (i.e., the average or the minimum and maximum filter lengths) are main parameters that can affect final detection results. To justify the robustness of the proposed method (and its variants), a set of parameter values is selected on the basis of the largest nucleus in our dataset. We obtain this size (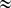 25 pixels) from a 3D image having fewer objects using simple threshold method. For multiscale based methods (Proposed and Variant-2), the minimum and the maximum filter lengths (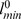 and 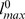) were varied from 5 to 50 and 17 to 62 pixels, respectively. A fixed interval (empirically chosen) of 12 pixels is maintained between each pair (minimum, maximum) of parameters (e.g., (5, 17)). A set of parameter pairs is thus computed applying linear sampling on both parameter ranges using 5 pixels interval. Filter lengths for multiscale filtering (spatial) within each pair is obtained using a fixed sampling interval, 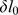, of 3 pixels (Eq. 7). For single-scale based methods (Variant-1 and Variant-3), initial filter length (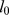) is varied from 5 to 50 pixels with an increment of 5 pixels.

**Figure 14:**
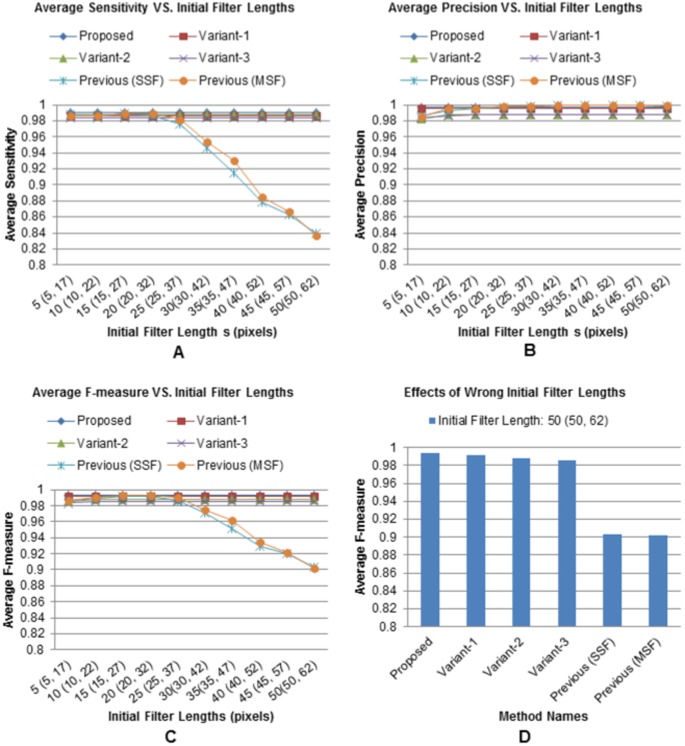
Robustness against parameter variations. Time average scores of (A) sensitivity, (B) precision, and (C) F-measure against the variations of initial filter parameters; and (D) average F-measure scores, obtained by the proposed method and its variants, when initial filter parameter(s) are randomly selected to high values, e.g., (50, 62) pixels for multiscale and 50 pixels for single-scale filtering.

**Figure 15:**
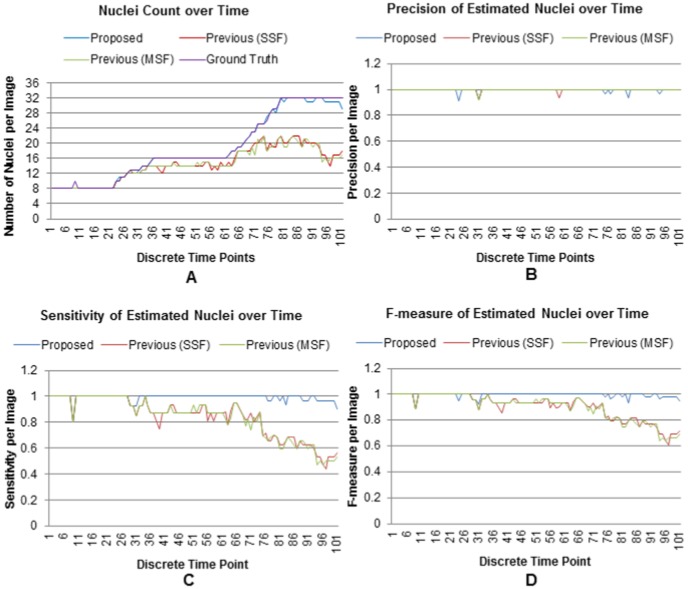
Robustness against high-valued parameters. (A) The number of estimated nuclei and the scores of (B) precision, and (C) sensitivity, and (D) F-measure over time, when arbitrarily large parameter values (e.g., (50, 62) pixels for multiscale, and 50 pixels for single-scale) are selected.

Analysis results were shown in Fig. 14, where blue, red, green, and purple curves show variations of sensitivity, precision, and F-measure metrics for the proposed method and its variants. Clearly, all three metrics for our adaptive methods preserve high accuracy (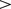 98 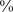) despite having a large variations of filter parameters. However, sensitivity (Fig. 14(A)) and F-measure (Fig. 14(C)) curves of the previous methods start dropping at (25,37) (multiscale) and 25 (single-scale) and ultimately reach to the 83.71 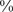 and 90.20 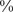, when filter parameters reach to the high end of the selected range. Above observation is re-confirmed by the F-measure plots at the highest parameter settings (Fig. 14(D)), when previous methods drop to 90.20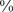 and 90.35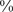, while the proposed method and its three variations maintain their high detection rates, i.e., 99.31 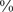, 99.13 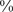, 98.77 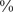, and 98.56 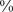, respectively. Precisions for all methods remain in the high states as expected because over-smoothing by large filter lengths reduces both the true and the estimated nuclei without increasing false detections (Fig. 14(B)). Performance degradation due to the wrong selection of filter parameters can be well explained by Fig. 15. All metrics (i.e., nuclei-count, sensitivity, and F-measure) except precision (red and green lines) start dropping at t31 and never return to the high state for previous methods (Fig. 15 A, C, D). In contrast, the proposed method and its variants (blue lines) always maintain high metric values except slight drifts at higher time-points. Precision remains in the high state for all methods except some minor variations.

#### Robustness to External Noise

In order to justify the noise tolerance of the proposed method, additive white Gaussian noise is added to a 3D image before applying nuclear detection algorithm. The considered image is a snapshot of a developing embryo at the early blastocyst stage containing 32 nuclei. If 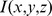 is the image and 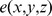 is the noise signal, generated from the noise spread 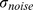, then signal-to-noise ratio (SNR) in dB and the noisy image (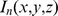) are obtained [30] by 
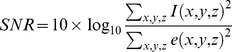
(19)

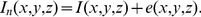
(20)


**Figure 16:**
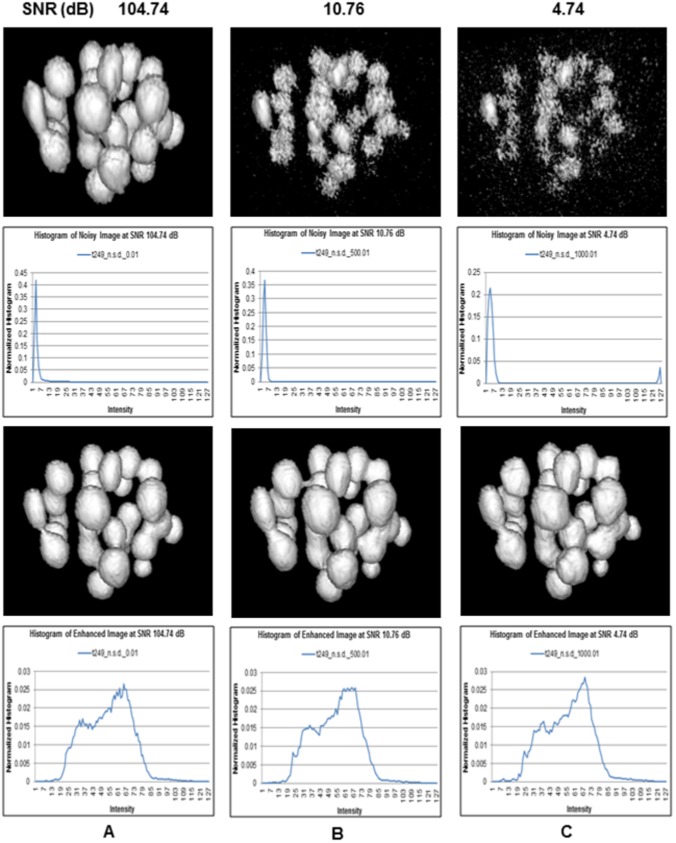
Robustness analysis against additive white Gaussian noise. Noisy image, its histogram, the enhanced image, and its histograms, when SNR levels are (A) 104.74 dB, (B) 10.76 dB, and (C) 4.74 dB, respectively. Proposed method recovers nuclei structures using the optimized initial parameters, i.e., 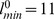, 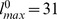, and 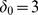 (pixel unit). A 3D image having 32 nuclei is considered for analysis.

To cover a large SNR range, i.e., 104.74–4.74 dB, we compute 30 noise spread values (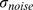) linearly using 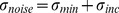, where 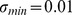 for the whole set of values, while 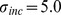 for the first 21 and 100.0 for the last 9 values. Our proposed method is then applied to the noisy image (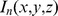) to recover nuclei objects. A sample result is shown in Fig. 16, where each column represents a noise (SNR) level, noisy image, its histogram, corresponding enhanced image, and its histogram, respectively.

**Figure 17:**
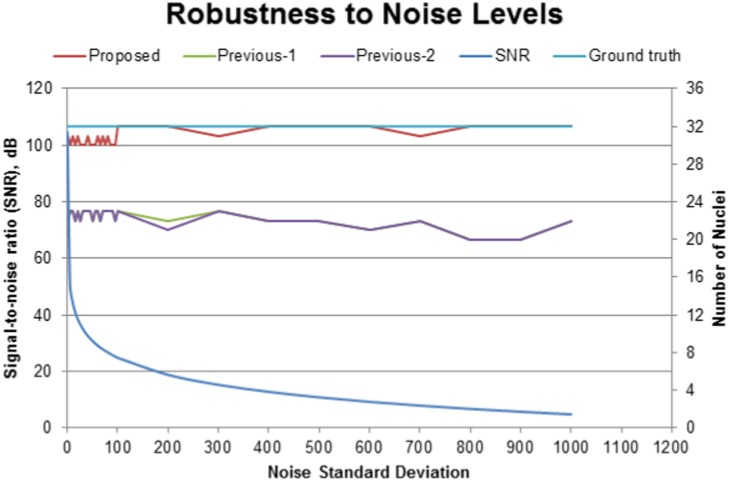
Robustness analysis against additive white Gaussian noise. (blue) SNR levels, (light-blue) ground truth, and the number of estimated nuclei for (red) the proposed, (green) previous-1, (purple) previous-2 against increasing noise spreads, when large initial parameters (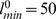, 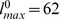, and 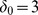) are selected. Note that the proposed method re-estimates a new set of parameters: 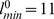, 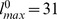, and 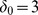 using feedback mechanism, while previous methods fail to do. A 3D image having 32 nuclei is considered for analysis.

First row shows that the image is gradually degraded with increasing noise variances, i.e., the decreasing SNR levels. Nuclear structures get quite damaged at the highest noise level, i.e., at 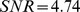 dB. Histograms in the second row indicate that the widths of the intensity distributions are expanding slowly with decreasing bin frequencies at increasing noise levels. This is what we expected since Gaussian noise randomly adds discrete black and white dots, which generates new intensities by breaking original intensity structures. However, our robust method recovers all objects by the adaptive multiscale filtering (Please refer to the third row of Fig. 16). Fourth row shows the histograms of the enhanced images that are almost same in structures, indicating the robustness of our method irrespective of the noise levels in the input images. Figure 17 shows the full experimental results, which include the graphs for SNR levels, ground truth nuclei numbers (light blue), and the estimated nuclei against the noise spread values. This figure shows that the selection of wrong parameters to large values (e.g., 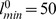 and 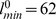) degrades the detection performances of the previous methods when cell population increases to 32 (green and purple lines). Note that the detection accuracy falls below 75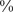, i.e., less than 24 nuclei are detected out of 32 nuclei, which further decreases due to increasing noise spreads (Fig. 17). In contrast, the proposed adaptive method quickly re-estimate the required parameters to (11,31) at 32 cell stage even though the initial parameters for the first frame were large, i.e., (50, 62); It achieves high detection accuracy 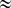 100 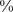, which remains almost steady irrespective of the noise levels in the input image (red line). This is the beauty of the proposed method because its multiscale spatio-temporal adaptive filtering recovers almost all nuclei through optimal enhancement.

Comparative analysis is summarized in the Table 1. Proposed method and Variant-1 show steady high F-measure (99.31 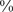, 99.13 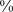) for all sets of initial filter lengths (Columns 2 and 3). F-measure metric values for Variant-2 and Variant-3 are smaller at the lowest end (98.47 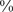, 98.32 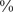) than those at the highest end (98.77 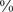, 98.56 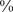) of the parameter range (i.e., (5,17) 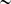 (50, 62) for MSF and (5 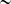 50) for SSF) indicating their sensitivity to small filter lengths. On the other hand, F-measure scores of Previous-1 and Previous-2 fall drastically with increasing filter lengths from their high values (99.30 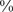, 99.20 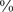) to the lowest values (90.20 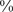, 90.35 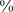), indicated as boldface numbers in the table. At this condition, our proposed method can achieve 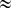 9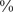 improvement over the previous methods. Note that the previous methods can also achieve good accuracies (99.30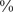, 99.20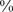) if the filter lengths are properly set to 15 for single-scale and (20,32) for multiscale cases (boldface numbers in the table). The robustness of the proposed method (and its variants) is due to the fact that the feedback mechanism computes proper filter lengths, which ensure accurate enhancement of the preprocessed image. As a result, unwanted local peaks even within nuclear region get suppressed and homogeneous objects are produced. In the non-adaptive case, there is no mechanism that can revert unexpected structures induced due to the improper enhancement.

**Table 1:** Summary of comparative performances based on F-measure metric.

Filter Lengths	Average (over time) F-measure ( 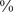 )					
single-scale (multiscale)	Proposed	Variant-1	Variant-2	Variant-3	Previous-1	Previous-2
5(5,17)	99.31	99.13	98.47	98.32	98.59	98.70
10(10,22)	99.31	99.13	98.77	98.50	99.06	98.92
15(15,27)	99.31	99.13	98.77	98.56	99.26	99.20
20(20,32)	99.31	99.13	98.77	98.56	99.30	99.12
25(25,37)	99.31	99.13	98.77	98.56	98.99	98.66
30(30,42)	99.31	99.13	98.77	98.56	97.48	97.03
35(35,47)	99.31	99.13	98.77	98.56	96.12	95.15
40(40,52)	99.31	99.13	98.77	98.56	93.39	92.93
45(45,57)	99.31	99.13	98.77	98.56	92.10	91.96
50(50,62)	**99.31**	**99.13**	**98.77**	**98.56**	**90.20**	**90.35**
Grand average	99.31	99.13	98.74	98.53	96.45	96.20
(over time and parameter space)						

Initial lengths for the enhancement filtering (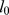, 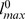, 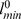) are varied in the range: (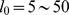) for single-scale, and (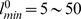; 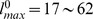) for multiscale filtering. Intermediate values for each parameter are obtained by linear sampling at an interval of 5, i.e., at 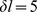. All parameters are in pixel unit.

## Discussion

A multiscale adaptive filtering based method is proposed for the robust detection of nuclei centroids from 4D fluorescence images. Three variants, namely a simplified version of the proposed method and single-scale versions of both methods, are also discussed. The use of feedback mechanism ensures appropriate enhancement and makes the proposed method robust against variations of filtering parameters and additive noise. Proposed method and its variants can produce more than 98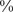 accuracy in the nuclei detection in terms of sensitivity, precision, and F-measure. Currently, we apply Gaussian and median filters for noise suppression. A better noise pre-filtering may further improve the detection results. Although simplified methods require less parameters, they (Variant-2 and Variant-3) may miss low contrast and small nuclei due to inappropriate selection of the minimum size threshold (i.e., 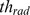) at the final stage of detection. In contrast, the proposed method performs threshold operation at an intermediate stage (i.e., at Stage-1), which may over-smooth the next sequential image if there are missing nuclei. However, the effect may not be severe due to a small number of missing nuclei, which can cause only a minor increase in the estimated parameters (e.g., minimum-, maximum- or average- nuclear diameter).

Proposed method requires the minimum and maximum lengths for the first frame only, while the rest of the frames in the time-series are automatically processed using feedback mechanism. The selection of these lengths may be based on the empirical knowledge about nucleus size. Since robustness analysis confirms the steady detection performance over a large range of parameter values (e.g., 10 times of the lowest parameter values as in Fig. 14), we can select the initial parameters by intuition. More precisely, we can estimate them by implementing a typical segmentation (e.g., manual/automated threshold) method to an image having fewer objects [22]. Free software (e.g., ImageJ) can also be used for this purpose [27]. Approximate size of a nucleus can then be used for selecting above parameters. Note that we feedback estimated minimum and maximum nuclei diameters as filter lengths for multiscale Gaussian filtering in the proposed method. Experimental analysis (not shown) also reveals that if we use cube filters instead of Gaussian, the feedback parameters have to be half of the estimated lengths for having the best detection accuracy.

Another observation is that multiscale enhancement-based method always performs better than its single-scale counterpart (Figs. 10 and 11). The variation of nuclei sizes (Fig. 12) during embryo development also suggests the use of multiscale filtering. However, the proposed multiscale based method showed 1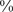 F-measure improvement over its single-scale version using the current dataset (Please refer to the last row of Table 1). The detail analysis as in Fig. 10 indicates that our method performs the most stable (i.e., less fluctuations) and the highest detection compared to all other variants. This observation also postulate that the proposed method may produce high detection accuracy in the densely populated scenario, e.g., neural or cancerous cells with large size variations.

Comparative study showed that the proposed method outperforms our previous method [18] and its single-scale variant. It can achieve approximately 9 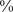 improvement compared to the previous methods, when filter parameter(s) are poorly selected to large values (i.e., 50 for single-scale, and (50, 62) for multiscale filtering)(Table 1).

Another advantage of our method is that we can segment nuclear volumes from clustered nuclei objects using the same feedback mechanism with a typical segmentation method (e.g., Local/Global threshold) (Fig. 8). This helps us analyzing nuclei dynamics through tracking nuclei over time. Nuclear volumes can also contribute to other applications like mitosis detection, cell cycle analysis, and phenotype clustering.

The decline of the nuclei detection accuracy at or after 32 cell stage happens due to decrease in RNA molecules over development as well as imaging limitations. We injected mRNA of H2B histone at one cell stage before the start of imaging procedure. Since RNA level per nucleus gradually decays with mitosis over time, the contrast of the image also decreases. Moreover, the received fluorescent signal along the z-direction decreases at all time-points due to scattering of the signal from deeper levels of the embryo. This causes non-uniform contrast along z-direction, which might be more pronounced when nuclear population increases at high time-points. In our analysis, we used imaging data up to the early blastocyst stage of mouse embryo development. An improvement in the detection accuracy at the late blastocyst stage may be possible using transgenic mice, which maintains approximately uniform intensity of fluorescent signal over time.

In this work, we did not perform any deconvolution operation on 3D/4D imaging data prior to image analysis. Instead, we performed simple noise filtering on our datasets using 3D Gaussian and median filtering. For early stage of development, deconvolution is not necessary because it may enhance micro-structures within nuclei that we suppress by Gaussian filtering. Applying multiscale filtering, rather than deconvolution, seems reasonable as the multiscale enhancement step of the proposed algorithm also takes care of scale of the typical objects and the noise levels in the imaging data. When high precision is expected, deconvolution operation might be considered especially for late developmental state. However, its outcome is uncertain without precise information about point spread function and noise characteristics of the confocal microscopy images. Iterative blind deconvolution is also expensive in case of processing 4D images and may cause artifacts. For any absolute demand, an efficient and accurate deconvolution technique need to be developed for processing high dimensional images.

Our method for automated extraction of nuclear information from 3D images can be a useful tool for understanding cell therapy processes. In this paper, we achieved about 99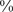 accuracy of detection compared with our previous results with 90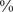 accuracy. In tracking of embryogenesis, we have to detect many nuclei in 4D images. For example, our data consists of about 270 time frames. If each frame contains 10 embryos on average, we have to detect 2160 nuclei. With 99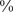 accuracy, we will have 21 erroneous nuclei that should be corrected manually or by other methods, whereas we have 210 erroneous ones with only 90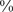 accuracy of our previous method. 21 is reasonable enough for manual correction but 210 is too much. In this respect, our method is relevant for the application of mouse embryogenesis tracking. Moreover, our proposed segmentation method can be extended to extract intra-embryo regions at early blastocyst stage; for example, the volume ratio of inner-cell-mass and blastocoel regions is a useful parameter for embryo quality assessment in the In-Vitro-Fertilization (IVF) treatment. By employing new datasets specialized for these applications, we believe that our method will play pivotal roles.

## Conclusion

A robust method and its three variations are proposed for automated extraction of nuclei centroids from 3D fluorescence image sequences. The robustness in the nuclei detection is achieved by adaptively estimating filter parameters, which ensure proper enhancement of the preprocessed image for efficient nuclei detection. An optimization technique, employing absolute radial gradient as an objective function, is proposed to estimate these parameters. Finally, a centroid-driven segmentation method, employing the same optimization technique, is proposed to segment nuclear volumes. Experimental evaluation was performed on five image-sequences from five developing mouse embryos. Qualitative analysis showed promising results for the nuclei detection, volume estimation, and tracking. A detail quantitative analysis with 101 3D sequential single-embryo images showed very high detection performance (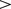
98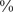) of the proposed method and its variants in terms of sensitivity, precision, and F-measure. Robustness analyses also showed very high and steady detection performances of the proposed methods irrespective of large variations of filter parameters and SNR levels with additive white Gaussian noise. Our analysis also showed that the proposed multiscale based robust methods are superior to their single scale counterparts. In future, the proposed methods will be extended for elaborate tracking experiments with more 3D embryo sequences involving cell division event.
